# Cases in Cook Co. Hospital

**Published:** 1870-11

**Authors:** C. T. Fenn


[The following was received too late for insertion in the usual place. Ed.]

***Cook Co. Hospital.*** Reported by C. T. Fenn, M.D.

Clinics are conducted in the Cook County Hospital with wonted regularity and interest. The following are notes of exercises in the service of Professors Ross, Powell, Johnson, and others of the attending staff:

Oct. 2. *Medical.* Ross. Case I. Albuminuria.—The presence of albumen in the urine tells us that we have a disease of the kidneys: congestion, or inflammation, or degeneration. There is failure of secretion of water and of elimination of urea. Hence we have dropsy and poisoning of the blood. *Treatment*—1. Subdue inflammation. 2. Overcome dropsy. 3. Eliminate urea. Apply fomentations to the back, sometimes cupping, if the inflammation be acute. Keep the patient quiet and warmly covered in bed; give him bland diet. Excite free diaphoretic action; the best method is by the hot air bath; a gallon of water may thus be obtained at once. You may eliminate water by the bowels. I would not use elaterium. One of the best means is bitartrate of potash, combined with a little jalap. This seems also to have a mild effect on the kidneys as a diuretic. Such use of medicine is opposed by the testimony of many high names, still, we favor it. This also affords elimination of urea.

Case II. A blacksmith, two weeks ago, taken down with a chill and pain in the joints. On admission he had the appearance of typhoid fever, but there was no diarrhœa or tympanitis. He had high fever and a dry tongue. A large amount of effusion existed in the left wrist, and crepitus. The right knee has now a large amount of effusion. The diagnosis is obscured. It will not do to treat it as rheumatism or gout; but, in rheumatic gout, we may have a state of things analogous to this. *Treatment*—Quinine, in two grain doses, three or four times daily, with wine and Opium at night.

Case III. Fever. Admitted Oct. 10. For several days previously he had complained of headache, loss of appetite, and chills. He then took his bed. On admission, the symptoms were those of typhoid, viz.: febrile disturbance, diarrhœa, tympanitis, and gurgling in right iliac fossa. Stools the color of pea-soup—characteristic. [Stools exhibited.] *Treatment*—Hydrochoric acid, twenty drops, every two hours; and, to control diarrhœa, turpentine emulsion. Sponge the body with cool water daily. Give beef-tea and milk—a pint of beef-tea and two pints of milk daily.

Case IV. Fever. Like the above.

*Surgical.* Powell. Case I. Results in fracture of the thigh. 1. Stiffness of the knee. Every limb is useless for a long time after the union of bone. 2. Shortening. If you get an inch of shortening you have a good result. As a rule in the hospital, we get from one-fourth to one-half an inch shortening. Measure from the anterior superior spinous process of the ilium. We may be so fortunate as to get none. 3. Length of time dressings should remain. Keep them on ten weeks, as a rule. Dressings must be as simple as possible. A weight and pulley for fractures of the femur, and a couple of bags of bran to keep the leg steady. There is one objection only to the weight and pulley; it may be the knee is left a little stiffer by their use. As a rule, don't think your patient will be able to walk, or work with ease, under six months. 4. Callus. As a rule, if you find that a large amount of bone has formed around the end of a fragment, you may know the bones have not been kept still.

CASE II. Colles's fracture. The most frequent form of fracture. A woman, washing windows, fell from the second story to the ground. To detect deformity, run the eye over the case. Charges of malpractice are most frequent in this form of injury. It is safe to say that the movements of the wrist will always be impaired. There will be a peculiar deformity. The diagnosis cannot be mistaken. Remember that you can hardly ever have a fracture of the lower end of the ulna. *Treatment*—Don't put a bandage next the skin. Never use a soft splint, as pasteboard. No evaporating lotions. You should have some pieces of board. Fit two to the well arm. They should not extend to the fingers. They should be a little wider than the arm. Pad them with sheet batting. In country practice, an old sheet is best, six or eight thicknesses. You may correct deformity by a compress. Then take hold, and make extension and counter-extension. Take off splint in six weeks, not sooner. A pistol splint does no good. But, with a bad result, the old authorities might be cited to prove that you had not done all. See your case every three or four days. Look at the arm after the second day, to see that the bones are coapted. Afterward, avoid too frequent handling, for fear of delayed union.

CASE III. Woman. Result of a fracture of this kind, five weeks standing. It ought to be united. Sometimes you hear of dislocation of the radius outward; there is no such thing; but the appearance is due to a little shortening. Here there is no provisional callus, because the bones have been kept still. She will complain of tenderness and stiff fingers. This case seems perfectly united. You may remove the splint and rub the joints with some stimulating liniment. I advise you to make your own splints. With adhesive plaster, an old splint or a board, you may treat any conceivable fracture. A patent splint will not fit many different cases. Keep patent splints in your front office, if you please; they are for ornament merely.

CASE IV. Woman, with hand bitten by a man. The bite on her finger was followed by erysipelas, very painful, and resulting in an ulcer which may go down to the tendons, on the back of the hand. Object to be attained: relief of pain. Give Opium and Quinine. The effect of Opium, as an anodyne, is different when rubbed up with Quinine. I do not, ordinarily, believe in poultices; but the best thing here would be a poultice, to hasten a separation of the slough.

CASE V. Man, slightly wounded on the joint of a finger six weeks ago. The whole hand and arm has been immensely swollen. Erysipelas may attack every tissue and destroy it here, it has caused entire destruction of the metacarpal bone of the thumb. Do not treat it as though it were an ordinary inflammation. (Ladies retire.)

CASE VI. Shown to illustrate the ill effects of a long prepuce. Nature never intended that the prepuce should lie over the glans. A long prepuce is a nuisance, morally and surgically. The Jews never have cancer of the penis. Do not confound this balanitic inflammation from acrid discharges from impure women, with chancre or chancroid. Be careful that urine is not retained. If it is so, slit. Use carbolized water; no mercury, no medicine of any kind, but we may use evaporating lotions. Be especially careful in the diagnosis of all venereal diseases before commencing the use of mercury. But when disease makes its appearance from three to six months after an exposure, you may begin to use the protiodide in one-half grain doses three times daily. Continue it till all symptoms disappear. But the patient is not then well. He may have a second attack: treat in the same way. If a third attack, use Iodide or Bromide of Potassium. If you begin in every suspected case, you may induce the symptoms by mercury in their worst form, viz., sore mouth, falling of the hair—in a word, all that is most dreaded in syphilis.

CASE VII. Result, in cure of hydrocele of the cord, by injection. No change from normal condition to be seen. Do not cut these cases.

CASE VIII. Child. Hip joint disease. Wearing Sayer's splint. Object: to take weight of body off the joint.

